# Physical activity and mental health during the COVID-19 pandemic among individuals with amputation

**DOI:** 10.1371/journal.pone.0283762

**Published:** 2023-05-25

**Authors:** Kyle R. Leister, Kevin Heffernan, Taavy Miller, Tiago Barreira

**Affiliations:** 1 Department of Exercise Science, David B Falk College of Sport and Human Dynamics, Syracuse University, Syracuse, New York, United States of America; 2 Department of Clinical and Scientific Affairs, Hanger Clinic, Austin, Texas, United States of America; Albanian University, ALBANIA

## Abstract

The isolating nature of various COVID-19 mandates may have reduced physical activity (PA) and increased mental health symptomology among individuals with amputation. However, an investigation of mental health across PA levels before and after the onset of COVID-19 among this group has not been conducted. Therefore, the objective of this study was to investigate group differences in depression, anxiety, and post-traumatic stress symptomology among individuals with amputation who reported being physically “active,” “somewhat active,” or “inactivate” before and during the pandemic. Individuals with an amputation at any level (n = 91; 51% female; age = 52.5±15.5) completed an online questionnaire to assess demographic information, PA levels, and mental health throughout the pandemic. Group differences in self-reported PA before and after COVID-19 onset were assessed by the PA Guidelines for Americans recommendations. The Center for Epidemiologic Studies Depression Scale (CES-D), Generalized Anxiety Disorder (GAD-7), and Posttraumatic Stress Disorder Checklist (PCL-5) scales were used to assess group differences in mental health status. Before and after the onset of COVID-19, 33% and 42.9% of respondents reported that they were inactive, respectively. 58.2% of respondents reported decreased PA since the pandemic’s onset. Prior to the pandemic, active individuals reported lower CES-D (14.21 vs. 19.07; Cohen’s d: -0.414), GAD-7 (3.82 vs. 5.47; Cohen’s d: -0.359), and PCL-5 (15.92 vs. 21.03; Cohen’s d: -0.319) scores compared to inactive individuals. After the onset of COVID-19, scores remained lower for active respondents CES-D (12.67 vs. 20.03; Cohen’s d: 0.-669), GAD-7 (3.17 vs. 5.87; Cohen’s d: -0.598), and PCL-5 (13.39 vs. 19.90; Cohen’s d: -0.430). Individuals with amputation reported decreased PA after the onset of COVID-19. Individuals reporting that they were “active” exhibited improved depression and anxiety symptomology scores compared to those reporting that they were “inactive.”

## Introduction

Various factors have exacerbated pre-pandemic barriers and produced novel barriers to physical activity (PA) for individuals with and without amputation during the COVID-19 pandemic. In the United States (U.S.), stay at home protocols to decelerate COVID-19 transmission may have represented one such novel barrier [1]. With lockdown measures in place, daily routines were disrupted and access to customary forms of exercise became more limited [2]. Specifically, gyms and fitness centers throughout the U.S. were instructed to shut down during the early phases of COVID-19. Additionally, many social activities possessing a physical component (walking groups, group fitness classes, etc.) were temporarily discontinued as people were urged to practice social distancing. As such, numerous studies reported overall reductions in PA during the pandemic in the general population [3, 4]. The implications of this are especially problematic for individuals with amputation, who frequently fell short of PA recommendations prior to the onset of the pandemic [5].

Adopting an active lifestyle can improve the physical and mental health of healthy and disabled populations [6–8]. Regular PA can improve body composition, glucose homeostasis, insulin sensitivity, and cognitive function [8]. In addition, increased PA has been shown to reduce C-reactive protein levels, which are inflammatory mediators that are elevated in individuals diagnosed with COVID-19 and many other chronic diseases [9–11]. Among individuals with existing cardiometabolic diseases including type 2 diabetes and coronary artery disease, sufficient PA can increase vascular function, potentially resulting in a shear-stress-mediated improvement in endothelial dysfunction [12]. Moreover, frequent PA positively impacts mental health by reducing stress, anxiety, and depression [13, 14]. Considering the wide array of health-related benefits associated with PA, an investigation into the impact of COVID-19 on PA levels among individuals with amputation is justified. Before the onset of COVID-19, many individuals with amputation struggled to meet PA recommendations [6, 7]. Studies have reported that individuals with amputation are often sedentary [15]. Additionally, this population has largely performed poorly on clinical-based PA measures including the timed up and go, and the 6- and 2-minute walk tests [16]. Psychological factors including fear of falling, body image anxiety, and lack of social support may also influence an individual with amputation’s ability to meet daily PA goals [16].

COVID-19 stay at home orders may have also contributed to mental health declines among this population. Research pertaining to COVID-19’s influence on mental health has focused on the effects of isolation, quarantine, and digital technology usage on various populations and psychological conditions [17–19]. Increased isolation associated with several COVID-19 restrictions has resulted in altered daily behaviors and contributed to elevated levels of stress and depression in vulnerable groups [2, 11]. With lockdown measures in place, digital technology utilization has increased, resulting in decrements in mental health and PA. Arora et al. reported that COVID-19 lockdowns may have contributed to drastic increases in unhealthy usage of digital technologies including smartphones, social media, and internet [18]. In this study, participants confined to their home were found to demonstrate addictive tendencies towards their digital medial usage, with increased usage equating to feelings of sadness and anger. Similarly, a second study featuring over 200 Twitter users found that individuals who spent extended periods on the platform were largely angry, disgusted, or sad and exhibited addictive behaviors pertaining to their overall Tweeting habits [19]. The consequences of increased digital technology usage have manifested in decreased mental health and emotional well-being [18].

While these connections are consistent with the general population, social isolation can have a particularly negative effect on individuals with amputation. Because of mobility limitations, individuals with amputation often rely on others for in-person visits, which may limit their social network [20]. Furthermore, evidence suggests that before the pandemic, social anxiety, isolation, and depression were common among this group, especially immediately after amputation surgery [21].

Self-reported PA level differences before and after the onset of COVID-19 have not been examined among individuals with amputation. Furthermore, mental health status across PA levels has not been compared among this group. Given the importance of PA on physical and mental health, these relationships merit investigation.

As such, the specific aims of this study were to: 1) examine concerns pertaining to health care accessibility, isolation, and social distancing practices during COVID-19 and 2) to investigate group differences in depression, anxiety, and post-traumatic stress symptomology among respondents who reported that they were physically “active,” “somewhat active,” or “inactivate” before and after the onset of the pandemic.

To address these aims, demographics, PA, and mental health status were assessed. It was expected that individuals with amputation may have experienced increased health care accessibility challenges and isolation during the pandemic. It was also hypothesized that individuals reporting inactivity during the pandemic would present higher mental health symptomology compared to those reporting that they remained “active” during the pandemic.

## Materials and methods

### Participants

Survey data were collected between November 2020, and April 2021, which was after the onset of the pandemic. The survey was comprised of questions pertaining to the participant’s experiences and PA status before the COVID-19 pandemic began. The same survey then asked the participant questions about their experiences and PA levels more recently during the pandemic. Because survey data were collected after the onset of the pandemic, all participants were required to retrospectively recall aspects of their PA and daily activities prior to the onset of COVID-19, and then compare potential changes that may have during the pandemic. Participants throughout the U.S. were recruited through online support groups, prosthetic component manufacturer social medial platforms, and the Amputee Coalition website. Individuals with amputation were also asked to complete the survey during prosthetic-related appointments at prosthetic clinics across the U.S. All participation was voluntary, and the survey could be accessed by scanning a QR code or a link from a virtual or paper flyer.

All participants provided electronic consent prior to completing the survey and were over the age of 18. Participants could have an amputation at any level (upper or lower extremity) but must have been using a prosthesis at least part time. All questionnaires were administered via Research Electronic Data Capture (REDCap) and the research protocol was approved by the Syracuse University Institutional Review Board (IRB).

### Scales

Participants were required to complete seven sections of questions. Section one consisted of demographic information. Demographic information was collected to determine sample characteristics including age, sex, level of amputation, years of prosthesis utilization, and medical history. Sections two through four consisted of established, clinically validated mental health scales [22–24]. Sections five through seven were comprised of COVID-19-specific PA and mental health questions. Questions in sections five through seven were specifically formulated to evaluate the participant’s experience during COVID-19.

Participants were prompted to retrospectively recall their PA level for questions pertaining to experiences before the onset of COVID-19. For questions pertaining to PA participation after the onset of COVID-19, participants were instructed to report their perceptions at the time of survey completion (present day).

#### Mental health scales

The Center for Epidemiologic Studies Depression Scale (CES-D), Generalized Anxiety Disorder (GAD-7), and Posttraumatic Stress Disorder Checklist civilian version (PCL-5) were used to assess depression, anxiety, and posttraumatic stress disorder (PTSD) symptoms, respectively [22–25]. CES-D scores >16 may be used to identify individuals at risk for clinical depression [26]. GAD-7 scores are divided into four categories: 0–4 indicate minimal anxiety; 5–9 indicate mild anxiety; 10–14 indicate moderate anxiety; and 15–21 indicate severe anxiety [24]. PCL-5 scores between 31–33 are indicative of PTSD [27]. In the current study, respondents were prompted to answer questions from all three scales based on their feelings, behaviors, and actions within the last week.

The CES-D and GAD-7 scales were found to have good reliability and criterion, construct, factorial, and procedural validity [28]. Both measures have been used in previous studies to assess generalized depression and anxiety among individuals with amputation [28–30]. The PCL-5 was also determined to be a psychometrically sound instrument, and has been used to assess PTSD symptoms after traumatic amputation [31].

#### COVID-19 specific questions

Sections five, six, and seven were comprised of questions requiring participants to reflect on their PA and personal concerns before and after the onset of COVID-19. For questions pertaining to specific phases of COVID-19 mandates, the pandemic was divided into three broad categories: 1) initial onset of the pandemic; 2) self-quarantine phase (no contact); 3) post quarantine (limited contact). Questions pertaining to personal concerns were established to examine the extent to which COVID-19 impacted financial situations (i.e., Regarding your current and near future financial situation, would you say that you are: Not worried at all; Worried if the COVID-19 changes last a few months; Worried about your retirement funds; Worried about paying bills in the near future; Very worried because you cannot afford to pay your current bills; Other/Prefer not to say), travel (i.e., Have you had to make changes to travel plans due to COVID-19 restrictions?: Yes; No), transportation (i.e., What has contributed most to the inability to access services?: Lack of transportation; Mobility limitations; Office restrictions/closures; Fear of COVID-19), social distancing (i.e., Are you currently: Mandated quarantine (staying completely isolated at home or hospital); self-isolation (staying isolated at home); Practicing strict social distancing (mostly alone/with family at home and trying to stay at least 6 feet away from others while outside the home); Practicing some social distancing (mostly alone/with family at home but not worried when going outside); Going about your normal life (going out without worry); Required to come in contact with others due to work; Other), and overall access to medical services (i.e., Since the onset of COVID-19 my ability to access medical services has: Improved; Remained the same; Worsened), both before and after the onset of COVID-19.

PA levels before and after the onset of COVID-19 were assessed with two questions where participants were asked to self-identify their perceived level of fitness based on PA guidelines for Americans [32]. Participants were first asked if they considered themselves “very active” (>300 minutes of moderate to vigorous PA a week), “active” (150–299 min of moderate to vigorous PA a week), “somewhat active” (75–149 min of moderate to vigorous PA a week), or “inactive” (<75 min of moderate to vigorous PA a week) before and after the onset of COVID-19 [32]. The “very active,” and “active” respondents were combined to a single group due to low representation in the “very active” category. Participants were also asked if they felt that their PA levels had increased, decreased, or stayed the same since the onset of COVID-19 (i.e., Since the onset of COVID-19, my level of physical activity has: Increased; Decreased; Remained the same).

### Data analysis

Data were imported into SAS (version 15; SAS Institute, Cary, NC, USA) for processing and to calculate scores for the GAD-7, CES-D and PCL-5. All data analyses were completed in SPSS (version 27; IBM Corp., Armonk, NY, USA).

Descriptive statistics (frequencies and proportions) were used to examine categorical variables. The effect size between self-reported “active” and “inactive” participants was determined Cohen’s d, interpreted as trivial (d = 0.0–0.19), small (d = 0.20–0.49), medium (d = 0.50–0.79), and large (d = 0.80 or higher) [33].

One-way analysis of variance (ANOVA) was used to determine group differences between pre- and post-pandemic self-reported PA levels (three groups: active, somewhat active, inactive) and CES-D, GAD-7, and PCL-5 scores. Subsequent analysis of covariance (ANCOVA) was completed to control for variability within and across the three PA levels before and after the onset of COVID-19. ANCOVA included covariates of lack transportation accessibility, financial concerns regarding paying bills, preexisting psychological problems, frequency of contact with others, and isolation status. Covariates were assessed for collinearity and selected using linear regression and stepwise selection functions in SPSS.

## Results

A total of 153 adults with amputation provided consent and began the questionnaire. Of this group, 62 did not complete all seven sections of the questionnaire and were excluded from data analysis. After exclusion, 91 respondents (52% female, 52.8±15.5 years) remained with complete data (Table 1). Over half (54.9%) of participants had an amputation at the transtibial (below knee) level. Most participants (39.6%) reported using a prosthesis for one to three years and cited trauma as the primary cause of amputation (37.4%). Most respondents (40.7%) were satisfied with their prosthesis, although some reported being “very satisfied,” (20.9%) or “dissatisfied,” (6%).

**Table 1:** Characteristics of individuals with amputation who participated in survey.

Demographic Characteristics	n	%
**Sex**		
Male	47	51.6%
Female	44	48.4%
**Amputation Level**		
Transtibial	50	54.9%
Transfemoral	21	23.1%
Transradial	10	10.0%
Transhumeral	2	2.2%
Bilateral/Multiple	8	8.8%
**Prosthesis Utilization**		
1–3 Years	36	39.6%
4–7 Years	22	24.2%
8–10 Years	8	8.8%
10+ Years	23	25.3%
**Prosthesis Satisfaction**		
Very Satisfied	19	20.9%
Satisfied	37	40.7%
Somewhat Satisfied	26	28.6%
Dissatisfied	6	6.6%
**Cause of Amputation**		
Trauma	34	37.4%
Congenital	4	4.4%
Type 2 Diabetes	17	18.7%
Cancer	11	12.1%
Dysvascular	7	7.7%
Other	17	18.7%
		**mean ± SD**
**Mental Health Scales**		
CES-D	91	16.85 ± 11.95
GAD-7	91	5.51 ± 4.93
PCL-5	91	17.56 ± 17.16

*Note*: Percentages may not add up to 100% due to missing data (participants declining to respond to certain measures). T-Scores, CES-D, GAD-7 and PCL-5 scores represented as (mean ± SD).

Abbreviations: CES-D: The Center for Epidemiologic Studies Depression Scale, GAD-7: Generalized Anxiety Disorder), PCL-5: Posttraumatic Stress Disorder Checklist.

Approximately 63% of respondents knew someone who had tested positive for COVID-19, and 13.2% of respondents had been diagnosed with COVID-19. Although 51.6% of participants did not experience decreased access to a prosthetist, 40.7% of respondents reported that they had become less likely to leave the house for basic needs (pharmacy, grocery store, etc.).

Nearly half (48.4%) of participants reported adhering to strict social distancing guidelines (i.e., reducing physical contact with other people). A quarter (24.2%) of participants reported practicing some social distancing, while only 4.4% of respondents reported going about life as usual. Despite these findings, 56% of the sample reported that their personal concerns regarding COVID-19 were equally severe during the initial onset, self-quarantine phase (no contact), and post quarantine (limited contact) periods of the pandemic.

In addition to high unemployment rates (45%) among the sample, 12.1% of employed respondents experienced reduced workload and salary because of COVID-19. Furthermore, 24% of respondents reported concern regarding their retirement funds and 26.4% reported being concerned about paying bills on time (Table 2).

**Table 2:** COVID-19-specific survey question responses.

COVID-19 Experience Questions	n	%
**COVID-19 status**		
Tested positive and recovered	12	13.2%
Personally knew someone who tested positive	58	63.7%
**Access to prosthetist**		
Experienced decreased access during the initial onset of the pandemic	16	17.6%
Self-quarantine (no contact)	21	23.1%
Post quarantine (limited contact)	3	3.3%
Never experienced decreased access	47	51.6%
**How likely were you to leave your house for basic needs?**		
Regularly	10	11%
Sporadically	40	44%
Rarely	37	40.7%
**Social distancing practices**		
Mandated quarantine	1	1.1%
Self-isolating	12	13.2%
Strict social distancing	44	48.4%
Some social distancing	22	24.2%
Going about normal life	4	4.4%
Required contact with others	4	4.4%
Other	2	2.2%
**Personal concerns relating to COVID-19**		
Initial onset of pandemic	10	11%
Self-quarantine (no contact)	11	12.1%
Post quarantine (limited contact)	6	6.6%
Equally concerned throughout	51	56%
Never concerned	10	11%
**Employment/workload during COVID-19**		
Increased workload	7	7.7%
Situation unchanged	17	18.7%
Reduced workload but maintained salary	1	1.1%
Reduced workload and salary	11	12.1%
Now working from home	5	5.5%
Unemployed	41	45.1%
Other	4	4.4%
**Physical activity before onset of COVID-19**		
Active*	28	30.8%
Somewhat active	33	36.3%
Inactive	30	33%
**Physical activity after onset of COVID-19**		
Active*	18	19.8%
Somewhat active	34	37.4%
Inactive	39	42.9%

*Note*: Percentages may not add up to 100% due to missing data (participants declining to respond to certain measures). *“Very active” and “active” groups combined due to low responses in “very active” category.

### Physical activity

Before the onset of COVID-19, 33% of respondents reported that they considered themselves inactive. After the onset of COVID-19, 42.9% of respondents reported that they were inactive. Most participants (58.2%) reported that their level of PA had decreased since the beginning of the pandemic, with the most remarkable change occurring during the self-quarantine (phase 2) phase.

### Mental health

Regarding mental health, 48.5% of respondents reported changing habits or behaviors to improve mental health during the pandemic. Activities including reading (n = 22) and acquiring new hobbies (n = 21) represented the most popular methods for improving mental health. Participants reported feeling most isolated (45.1%) and experiencing the greatest change to their mental health (36.3%) during the self-quarantine (phase 2) of the pandemic. Mental health scores among PA levels before and after the onset of COVID-19 can be found in Table 3.

**Table 3:** Means, standard deviations, and one-way ANOVA for mental health scores among PA levels before and after COVID-19 onset.

*Prior to COVID-19 onset*					*After onset of COVID-19*			
	n	Mean + SD	*F* (2,88)	95% CI	n	Mean ± SD	*F* (2,88)	95% CI
**CES-D–Depression Scale**			1.20				2.83	
Active*	28	14.21±12.87		[9.22,19.21]	18	12.67±10.73		[7.33,18.00]
Somewhat Active	33	17.06±12.30		[12.70,21.42]	34	15.41±12.82		[10.94,19.89]
Inactive	30	19.07±10.95		[15.14,22.99]	39	20.03±11.12		[16.42,23.63]
**GAD-7 –Anxiety Scale**			1.58				1.95	
Active*	28	3.82±3.86		[2.32,5.32]	18	3.17±3.48		[1.43,4.90]
Somewhat Active	33	6.00±5.41		[4.08,7.92]	34	5.38±5.42		[3.49,7.27]
Inactive	30	5.47±5.15		[3.54,7.39]	39	5.87±4.91		[4.28,7.47]
**PCL-5 –PTSD Scale**			0.94				0.90	
Active*	28	15.29±16.76		[8.79,21.79]	18	13.39±11.42		[7.71,19.07]
Somewhat Active	33	16.33±15.57		[10.81,21.86]	34	17.09±20.14		[10.06,24.12]
Inactive	30	21.03±19.11		[13.89,28.17]	39	19.90±16.51		[14.54,25.25]

*Note*: *“Very active” and “active” groups combined due to low responses in “very active” category.

### Physical activity and mental health before COVID-19 onset

Before the onset of COVID-19, one-way ANOVA did not reveal significant group differences in CES-D (F (2,88) = 1.206, p = 0.30, 95% CI [1.25, 14.36]), GAD-7 (F (2,88) = 1.589, p = 0.21, 95% CI [4.13, 6.18]), or PCL-5 (F (2,88) = 0.943, p = 0.39, 95% CI [13.99, 21.13]) symptomology scores across pre-COVID PA levels (three levels: active, somewhat active, inactive). Furthermore, small effect sizes were noted when comparing mental health measures (CES-D (14.21 vs. 19.07; Cohen’s d: -0.414), GAD-7 (3.82 vs. 5.47; Cohen’s d: -0.359), and PCL-5 (15.92 vs. 21.03; Cohen’s d: -0.319)) between “active” and “inactive” respondents.

#### CES-D and physical activity before COVID-19 onset

Stepwise linear regression determined that covariates of lack of transportation accessibility (p<0.001) and preexisting psychological problems (p<0.01) were significantly related to CES-D depression scores. Despite these findings, ANCOVA did not reveal significant group differences in depression symptomology scores (F (2,88) = 0.463, p = 0.63) across pre-COVID activity levels (three levels: active, somewhat active, inactive).

#### GAD-7 and physical activity before COVID-19 onset

Stepwise linear regression determined that covariates of lack transportation accessibility (p<0.001) and preexisting psychological problems (p<0.05) were found to be significantly related to GAD-7 anxiety scores. Despite these findings, ANCOVA did not reveal significant group differences in anxiety symptomology scores (F (2,88) = 1.11, p = 0.32) across pre-COVID activity levels.

#### PCL-5 and physical activity before COVID-19 onset

Stepwise linear regression determined that covariates of lack transportation accessibility (p<0.001) and preexisting psychological problems (p<0.05) were found to be significantly related to PCL-5 PTSD scores. Despite these findings, ANCOVA did not reveal a significant group difference in PTSD symptomology scores (F (2,88) = 0.132, p = 0.87) across pre-COVID activity levels.

### Physical activity and mental health after COVID-19 onset

After the onset of COVID-19, one-way ANOVA did not reveal a significant group difference in CES-D (F (2,88) = 2.83, p = 0.06, 95% CI [14.36, 19.34]), GAD-7 (F (2,88) = 1.95, p = 0.14, 95% CI [4.13, 6.18]), or PCL-5 (F (2,88) = 0.904, p = 0.40, 95% CI [13.99, 21.13]) symptomology scores across PA levels during COVID-19. Furthermore, medium effect sizes were noted when comparing the CES-D (12.67 vs. 20.03; Cohen’s d: 0.-669) and GAD-7 (3.17 vs. 5.87; Cohen’s d: -0.598) mental health measures and small effects sizes were noted when comparing the PCL-5 scale (13.39 vs. 19.90; Cohen’s d: -0.430) between “active” and “inactive” participants.

#### CES-D and physical activity after COVID-19 onset

Covariates of lack of transportation accessibility (p<0.001), financial concerns regarding paying bills (p<0.05) and preexisting psychological problems (p<0.05) were significantly related to CES-D depression scores. Despite these findings, ANCOVA did not reveal significant group differences in depression symptomology scores (F (2,88) = 1.39, p = 0.25) across activity levels during COVID-19.

#### GAD-7 and physical activity after COVID-19 onset

Covariates of lack of transportation accessibility (p<0.001), financial concerns regarding paying bills (p<0.05) and preexisting psychological problems (p<0.05) were significantly related to GAD-7 anxiety scores. Despite these findings, ANCOVA did not reveal significant group differences in anxiety symptomology scores (F (2,88) = 0.430, p = 0.65) across activity levels during COVID-19.

#### PCL-5 and physical activity after COVID-19 onset

Covariates of lack transportation accessibility (p<0.001), financial concerns regarding paying bills (p<0.05) and preexisting psychological problems (p<0.001) were found to be significantly related to PCL-5 scores. Despite these findings, ANCOVA did not reveal significant group differences in PTSD symptomology (F (2,88) = 0.081, p = 0.92) across activity levels during COVID-19.

## Discussion

This report presents data from an online survey examining personal concerns pertaining to health care accessibility, isolation, transportation, and social distancing before and after the onset of COVID-19. In addition, group differences in depression, anxiety, and post-traumatic stress symptomology were investigated among respondents who reported that they were physically “active,” “somewhat active,” or “inactivate” before and during the pandemic. Most participants reported decreased PA after the onset of COVID-19, especially during the pandemic’s initial lockdown and self-quarantine phases. Significant group differences in depression, anxiety, and PTSD symptomology were not noted across all three groups. Despite these findings, medium effect sizes were noted when comparing CES-D and GAD-7 scales between participants reporting that they were “active” versus “inactive” after the onset of the pandemic.

In the current study, 33% of respondents considered themselves inactive before the onset of COVID-19. After the onset of COVID-19, 42.9% of respondents reported increased inactivity, with the greatest changes in PA occurring in the self-quarantine phase. These findings are consistent with several studies investigating COVID-19-related changes in PA featuring healthy individuals [3, 34, 35]. In a study featuring 3,052 healthy adults, Meyer et al. reported a 32% decrease in self-reported PA among previously active individuals during COVID-19 [34]. A second study featuring over 1,000 electronic survey respondents reported a decrease in vigorous and moderate intensity exercise during stay at home mandates [3]. In addition to decreased PA, sitting times increased 28.6% during home confinement, largely due to quarantine, the inability to utilize fitness centers, and limitations on group recreational activities [3]. These results suggest that individuals with and without amputation had difficulty maintaining pre-pandemic levels of PA during the quarantine phase of COVID-19.

Mobility and environmental barriers may have contributed to PA declines during the pandemic. Before the onset of COVID-19 many individuals with amputation depended on family, public transportation, or taxis for transportation, which may have been further compromised during lockdown [36]. In the current study, the covariate “lack of transportation accessibility” was significantly related to mental health scores both prior to and after the onset of COVID-19. This finding suggests that individuals with amputation may have been unable to travel to areas where PA could potentially occur (parks, grocery stores, gyms, etc.). With decreased access to transportation, PA participation may have been limited to the home or neighborhood settings.

In addition to decreased transportation accessibility, the existence of preexisting psychological problems (operationally defined as any mental health disorder diagnosis before or after the onset of COVID-19) was also significantly related to mental health outcomes. Individuals with preexisting mental health conditions have reported higher anxiety and depression during the pandemic [37]. Furthermore, people with previous psychiatric illnesses were among the groups most affected by the COVID-19 pandemic [38]. During the initial onset of the pandemic, people were instructed not to leave their homes, which may have resulted in isolation. As such, individuals with preexisting psychological problems may have experienced difficulties obtaining medical assistance due to clinics reducing patient load to prevent COVID-19 spread in the hospital setting.

Depression, anxiety, and PTSD have been closely investigated throughout the pandemic, and numerous studies support that COVID-19 mandates have impacted mental health [39–42]. One study utilized the CES-D scale to assess depressive symptoms among over 10,000 participants [43]. Of the sample, a mean CES-D score of 16.7 was reported, indicating that many respondents were at risk for clinical depression [26]. In the current study, a mean CES-D score of 16.85 was noted among all respondents, also indicating clinical depression risk. Furthermore, participants that classified themselves as active had lower CES-D scores compared to respondents who classified themselves as inactive, which may support the notion that exercise and PA may ameliorate the effects of depression [44].

Concern over contracting COVID-19 and fear of adverse health reactions have contributed to elevated anxiety among the general population [45, 46]. In one large scale study, 71% of respondents reported some level of anxiety during the pandemic [45]. Furthermore, after categorizing anxiety as either “high” or “low” based on a GAD-7 cutoff of eight, nearly half of the respondents reported high anxiety throughout the pandemic. In contrast to these findings, a mean GAD-7 score of 5.5 was noted among individuals with amputation indicating mild anxiety.

GAD-7 scores in this study were comparable to pre-pandemic GAD-7 scores among individuals with amputation. Specifically, Ladlow et al. reported mean GAD-7 scores of 3 and between 2–4 in two different studies featuring U.K. military personnel who experienced amputation [29, 47]. Furthermore, a mean GAD-7 score of 6 was reported among a cohort of individuals who underwent amputation secondary to infection [48]. When comparing our findings with previous studies, anxiety levels did not differ during the COVID-19 pandemic. While unexpected, results from the current study remain consistent with other studies investigating anxiety among this population.

Increased PTSD symptomology has also been reported throughout the pandemic, especially among healthcare personnel, frontline workers, and individuals with severe COVID-19 symptoms [42, 49, 50]. Studies conducted prior to COVID-19 featuring individuals with amputation have reported that between 15% and 26% of individuals with amputation may experience PTSD [31, 51]. In the current study, mean PCL-5 scores among all respondents was 17.56, and 14.2% of respondents reported PCL-5 score >33, (a typical cut-point for PTSD) [27].

Participants who classified themselves as physically active reported lower PCL-5 scores before and the onset of the pandemic compared to those who reported that they were inactive. The relationship between PA and exercise has been investigated [52, 53]. A randomized controlled trial conducted by Mehling et al. found that participants with PTSD who were randomly assigned to a 12-week exercise program featuring aerobic and resistance training combined with yoga had positive effects on mindfulness and introspective awareness among individuals with PTSD [53]. A second study also found that vigorous-intensity exercise was inversely associated with PTSD symptomology [52]. Findings from these studies suggest that PA and exercise may play an important role in decreasing PTSD symptom severity in able-bodied individuals. However, very few studies have investigated the link between PTSD, exercise, and individuals with amputation. As such, findings from the current study may provide direction for future research in this area.

### Limitations

Several noteworthy limitations exist in the current study. Because the survey was distributed through various demographic regions across the U.S., interpretation of the specific COVID-19 phases may have varied depending on region and time of response. To ameliorate this limitation, a brief description of each phase was defined before the corresponding section of questions. Subject recruitment was executed through various social medial and online platforms, which may have reduced representation of individuals without internet access. However, opportunity to complete the survey was also provided during in-clinic prosthetic appointments. It should also be noted that the design of the study is cross-sectional yet discusses implications of the effect COVID-19 on changes in self-reported and retrospective measures of PA behavior. Finally, because the survey prompted participants to retrospectively reflect and report PA and mental health before the onset of COVID-19, retrospective self-recall bias may present. Despite these limitations, findings from this study provide insight for future research aimed at deciphering specific effects of isolation on mobility and mental health among individuals with amputation.

## Conclusions

Results from this study provide initial insight into the impact of COVID-19 on PA and mental health among individuals with amputation. Ultimately, there is some evidence to suggest that COVID-19 has contributed to PA decrements among individuals with amputation, as respondents largely indicated that their PA levels had declined during the pandemic. In all three mental health measures, more optimal mental health scores were noted among “active” compared to “inactive” individuals. These findings provide evidence that participating in PA may reduce mental health symptomology among this population.
